# The effects of vitamin D3 supplementation on TGF-β and IL-17 serum levels in migraineurs: post hoc analysis of a randomized clinical trial

**DOI:** 10.1186/s40780-021-00192-0

**Published:** 2021-03-03

**Authors:** Zeinab Ghorbani, Pegah Rafiee, Samaneh Haghighi, Soodeh Razeghi Jahromi, Mahmoud Djalali, Hedieh Moradi-Tabriz, Maryam Mahmoudi, Mansoureh Togha

**Affiliations:** 1 Cardiovascular Diseases Research Center, Department of Cardiology, Heshmat Hospital, School of Medicine, Guilan University of Medical Sciences, Rasht, Iran; 2 Department of Community Medicine, School of Medicine, Guilan University of Medical Sciences, Rasht, Iran; 3 Headache Department, Iranian Center of Neurological Research, Neuroscience Institute, Tehran University of Medical Sciences, Tehran, Iran; 4 Student Research Committee, Department and Faculty of Nutrition Sciences and Food Technology, Shahid Beheshti University of Medical Sciences, Tehran, Iran; 5 Headache Department, Neurology Ward, Sina University Hospital, School of Medicine, Tehran University of Medical Sciences, Tehran, Iran; 6 Department of Clinical Nutrition and Dietetics, Faculty of Nutrition and Food Technology, Shahid Beheshti University of Medical Sciences, Tehran, Iran; 7 Department of Cellular and Molecular Nutrition, School of Nutritional Sciences and Dietetics, Tehran University of Medical Sciences, Tehran, Iran; 8 Department of Pathology, Sina University Hospital, Tehran University of Medical Sciences, Tehran, Iran; 9 Pediatric Gastroenterology and Hepatology Research Center, Children’s Medical Center, Tehran University of Medical Sciences, Tehran, Iran; 10 Dietitians and Nutrition Experts Team (DiNET), Universal Scientific Education and Research Network (USERN), Tehran, Iran

**Keywords:** Cholecalciferol, Headache, Immune cells, Th17/Treg cytokines, Vitamin D3

## Abstract

**Background:**

Although the exact mechanism involved in migraine pathogenesis remained uncertain, and different researches have been developed to address the role of neuroinflammation and immune dysfunction. Therefore, considering the immune protective functions of vitamin D3, we aimed to investigate the effects of daily administration of 2000 IU D3 supplements on serum status of immune markers in migraine patients.

**Methods and materials:**

Eighty episodic migraineurs who randomly assigned into two equal groups to receive either vitamin D3 2000 IU/d or placebo for 12-week were enrolled in this placebo-controlled double-blind trial included. Serum concentrations of transforming growth factor-beta (TGF-β) and interleukin (IL)-17 were evaluated at baseline and after the trial via the ELISA method.

**Results:**

Applying ANCOVA adjusted for baseline levels and confounding variables, it was found that the serum level of TGF-β was significantly higher in vitamin D group (adjusted mean:1665.50 ng/L) than the placebo group (1361.90 ng/L) after the experiment (*P*-value = 0.012); on the other hand, vitamin D prevented the increment in IL-17 serum level in the intervention group after the trial (adjusted mean:37.84 ng/L) comparing to the controls (adjusted mean:70.09 ng/L; *P*-value = 0.039). The Pearson correlation analysis revealed a significant positive correlation between changes in serum 25-hydroxy-vitamin D (25(OH)D) and TGF-β (*r* = − 0.306, *P*-value = 0.008). In contrast, no significant correlations were noted between serum 25(OH) D and IL-17 changes throughout the study.

**Conclusion:**

Based on the results of this study, it was revealed that 12-week vitamin D3 supplementation (2000 IU/day) could enhance the Th17/Treg related cytokines balance in episodic migraineurs. Although these findings are promising, it is needed to be extended.

**Trial registration:**

The trial is registered in the Iranian registry of clinical trials (IRCT) at 11 July 2018, with IRCT code: IRCT20151128025267N6 (https://www.irct.ir/trial/31246).

## Background

According to the report of the global burden of disease (GBD) 2016, migraine headache has been established as the leading disability cause in the globe among under 50 years old [1]. It has been estimated that migraine affects about 11% of adults with a major impact on those aged between 25 to 55 years [1, 2], which consequently lead to high headache burden on individuals, communities, health care systems, and societies [3]. Migraine is 2–3 times more common in women than in men. Its attacks are usually more prolonged, with higher intensity and more disability among females [4]. Two main categories of migraine include episodic migraine (EM) (having < 15 headache days per month) and chronic migraine (CM) (having ≥15 headache days per month, of which at least 8 days are with migraine features for at least 3 months) [5]. If patients who suffer from episodic migraine, especially those with frequent attacks, are not treated appropriately, it can lead to chronic type, which is reported to be more severe with higher disability [6, 7].

Both genetics and the environment are supposed to be related to migraine development. Some dietary factors, misuse of caffeine, hormonal fluctuations, high-stress level, increased weight, smoking, and suffering from tension-type headache or medication overuse headache are among the suggested factors affecting migraine susceptibility and or development [8].

There is no established mechanism to explain migraine pathogenesis, although there have been many efforts directed in this field. The current evidence suggests a variety of mechanisms that are thought to be involved in the pathophysiology of migraine, including cortical spreading depression (CSD), trigeminovascular pathway activation (a pathway for nociceptive information conduction), neuroinflammation, and dysfunction in the vascular system [9, 10]. Different neuropeptides such as calcitonin gene-related peptide (CGRP) and pituitary adenylate cyclase-activating peptide (PACAP) with vasoactive properties are believed to be released during migraine attacks. The release of these peptides could affect the activation of the trigeminovascular system and subsequently might induce vasodilatation of arteries, plasma leakage, and mast cell degranulation [9]. Although the activation of mast cells during migraine attacks would give an insight into the role of immune dysfunction in migraine pathogenesis, more research is needed to explain the exact mechanisms related to the effects of immune cell imbalance in migraine [11–13].

There is a growing body of literature that recognizes the importance of T helper (Th) 17 cells and regulatory T cells (Treg) as two separate subsets of CD4+ T cells in the pathogenesis of a variety of inflammation-related disorders. Similar to Th1, the long-term presence of Th17 cells in a tissue can lead to the tissue destruction due to its pro-inflammatory effects and the recruitment of neutrophils to the tissue site. Recent evidence has suggested that Th17 cells may play an important role in conditions related to autoimmune disorders and inflammation. The main cytokine which produced by Th17 cells is interleukin (IL)-17 that comprises about six family members of related cytokines, of which the most prominent one is known as IL-17A. Other factors that are released by Th17 cells include IL-21 IL-23, IL-22, in addition to IL-6, tumor necrosis factor-α (TNF-α), and chemokines [11, 14–19].

Transforming growth factor-β (TGF-β) is believed to be a multi-characteristic factor that mostly known as a suppressive cytokine. It is of note that presence of TGF-β in addition to IL-6, IL-21, IL-1β, and IL-23, as well as the retinoid orphan receptor (RORγT), eventually result in promoting the differentiation of Th17 cells from naïve T cells [11, 14–19]. On the other hand, CD4+ T cells in the presence of TGF-β accompanied by IL-10, as well as the transcription factor forkhead box P3 (Foxp3), are differentiated to Treg cells. Treg cells are known with their immunosuppressive functions through producing anti-inflammatory cytokines such as IL10 as well as maintenance of self-tolerance mechanisms. IL-10 and TGF-β1 are the well-known Treg-related cytokines [11, 14–25]. However, to our knowledge, to date, little evidence has been found for the possible role of immune responses associated with Th17 and Treg cells in the pathogenesis of migraine.

Vitamin D has been reported to decrease serum concentrations of pro-inflammatory factors such as C-reactive protein (CRP), TNF-α, interferon-γ, and ILs-1β, 6, and 17 as indicators of inflammatory status. It also has been shown to elevate ILs-4, 5, and 10 and total antioxidant capacity, as markers of anti-inflammatory state [10, 12]. In addition to its anti-inflammatory effects, this vitamin is thought to modulate proliferation, differentiation, and activation of immune cells (i.e., macrophages) and immunological responses [26, 27]. Recovery of Th1/Th2 cells balance, inhibiting Th17 cell development and elevating less inflammatory Treg cells that produce regulatory mediators such as TGF-β, following vitamin D administration in experimental autoimmune encephalomyelitis (EAE), the animal model of multiple sclerosis (MS) has been highlighted before [28]. Interestingly, epidemiological studies suggest a negative influence of vitamin D deficiency in a variety of neurological disorders, including migraine [12, 29, 30].

We previously showed beneficial impacts of vitamin D on migraine headache frequency and its clinical features, through amelioration of CGRP serum levels and its anti-inflammatory effects [10, 31]. Most importantly, our previous findings from this trial delineated that vitamin D supplements could be useful in averting the inflammatory state in migraineurs through suppression of serum pro-inflammatory cytokines levels (IL-6, and inducible nitric oxide synthase (iNOS)). We also revealed that there might be negative correlations between changes in serum 25-hydroxy-vitamin D (25(OH)D) and these cytokines [10]. Therefore, it might be raised that vitamin D can also affect Th17/Treg cells balance, particularly due to regulatory role of IL-6 in this balance [32]; however, up to our knowledge, there is not any study concerning the immunological effects of supplementation with vitamin D in migraine headache. Thus, we aimed at further evaluating the efficacy of vitamin D as an immunomodulatory compound on the main cytokines related to Th17/Treg cells, including IL-17 and TGF-β within the same trial.

## Methods

### Participants and design

The information on the details of this trial and data collection procedure was described in our previous publications [10, 31]. This randomized, double-blind placebo-controlled trial included a 4-week baseline period followed by a 12-week intervention that was performed on 80 EM patients to investigate the effects of 2000 IU/d vitamin D supplement compared with placebo on headache characteristics and some serum biomarkers from July 2018 to July 2019 in the tertiary headache clinic of Sina University Hospital. The inclusion criteria were as follows: suffering from episodic migraine for at least 6 months before the trial on the basis of the study headache-specialists diagnosis in complying with the 3rd edition of International Classification of Headache Disorders (ICHDIII criteria) [33]; subjects should be between 18 to 45 years of age; and had body mass index (BMI) between 18.5–30 kg/m^2^. The exclusion criteria was as follows: those who had a diagnosis of medication overuse headache or other types of headaches in 3 months before the trial; consumed vitamin D supplements in 3 months before the trial; consumed other dietary supplements (i.e. magnesium, calcium, zinc, vitamin B groups or vitamin C), anti-epileptic, and anti-psychotic drugs, thiazide diuretics, glucocorticoid, statins or orlistat (e.g. topiramate, sodium valproate and carbamazepine) throughout the trial; the patients who were post-menopause, pregnant and lactating; the patients with a comorbidity of chronic disorders based on the past medical history and/or physician diagnosis (e.g. gastrointestinal disorders, liver and kidney dysfunction, sarcoidosis, rickets, osteomalacia and cancer); and those who did not intend to participate in or continue the present trial.

After initial screening, EM patients were randomized into vitamin D or placebo group (each consisted of 40 EM sufferers). All participants were allowed to consume their usual acute/ prophylactic medications provided that they were not changed during the study. The dosage of daily vitamin D supplementation was determined based on previous reports, which indicated 2000 IU of this vitamin could influence immune function and cytokine production from T helper cells [12, 34, 35]. Both vitamin D and placebo pearls were produced by Zahravi Pharmaceutical Company, Tabriz, Iran.

### Data collection

#### Headache diaries assessment

A 30-day headache diary, designed by the senior researcher Prof. M. T, and has been described in detail previously [36], was given to the included subjects to fill out the migraine headache characteristics during a month prior to the intervention initiation. Detailed findings regarding headache characteristics within this trial were reported in our previous paper [10].

#### Blood samples analysis

A five-ml blood sample was collected from all subjects at the first visit and the end of the intervention to explore serum concentrations of IL-17 and TGF-β using enzyme-linked immunosorbent assay (ELIZA) method via Crystal Day kit (Crystal day, China). The time interval from experiencing the last headache attack to the day of collecting blood samples was also asked.

#### Sample size calculation and statistical analysis

Based on below formula, it was assumed that if we aim to find at least 4 days decrease in the number of headache days per month and we include about 40 samples in each study arm (with a mean standard deviation (SD) headache day of about 8 (4) days per month), we would reach 90% statistical power (α = 0.05, β = 0.90, and S = 4 and d = 4) [37].

$$ n=2\frac{{\left({z}_{1-\frac{\alpha }{2}}+{z}_{1-\beta}\right)}^2S2}{(d)^2} $$

All statistical analyses were conducted via SPSS program version 24 (IBM Corporation, New York, USA). Normality of data was tested using Shapiro-Wilk test. Between-group comparisons were performed applying independent sample t-test and chi-square test in case of continuous and categorical data, respectively. To compare the effects of intervention or placebo on serum IL-17 and TGF-β levels, analysis of covariance (ANCOVA) adjusted for baseline levels and confounders was used and the adjusted means and 95% confidence intervals ​​(95% CI) were reported. Pearson correlation test was applied to explore the correlations between changes in serum 25(OH) D and these cytokines throughout the study. The figures were prepared using GraphPad Prism 5.0 (GraphPad Software, Inc.). Significance level was considered as *P* < 0.05 level.

## Results

Table 1 demonstrates the studied population baseline characteristics. This part of data are identical to our previously reported data [10, 31]. No significant differences in sex, age, headache onset years, and BMI were detected between the groups. The mean (SD) of age was 37 (8) and 38 (12) years in intervention and placebo arms of study, respectively. Around 80% of patients in each group were consisted of females (Table 1) [10, 31]. Besides, mean changes of serum 25 (OH) D levels from baseline through 12-week intervention in the vitamin D and placebo groups were + 12.34 ng/ml and − 0.28 ng/ml, respectively (mean levels after the trial: 39.65 ng/ml in vitamin D group and 33.95 ng/ml in placebo group) (*P* < 0.001) [10, 31].

**Table 1:** Baseline demographic, anthropometric and clinical data of episodic migraine patients in a randomized controlled trial of vitamin D vs. placebo

		Vitamin D (***n*** = 40)	Placebo (***n*** = 40)	***P*** value^**a**^
**Age (year)**		37 (8)	38 (12)	0.819
**Headache Onset years (year)**		12 (8)	11 (8)	0.679
**Body mass index (kg/m2)**		25.83 (4.07)	25.36 (4.29)	0.613
**Sex**	Female	32 (80.0%)	32 (80.0%)	1.000
	Male	8 (20.0%)	8 (20.0%)	

Values are expressed as mean (standard deviation (SD)) or number (%) as appropriate

^a^Using either Independent t test or Chi square according to the type of data

This data are identical to our previously reported data [10, 31]

Table 2 describes changes in the serum levels of cytokines related to Treg / Th17 immune cells following taking vitamin D supplements or placebo for 3 months. The mean time from headache last attack (to the day of blood samples collection at the end of study) was estimated as 5 and 4 days in vitamin D and placebo receiving patients, respectively. The independent sample t-test did not detect a significant difference between baseline mean values of TGF-β and IL-17 serum levels between the studied groups. Using ANCOVA controlled for baseline TGF-β levels, age, sex, BMI changes, years of having headache and number of days from the last headache attack showed significantly elevated serum levels of this cytokine in the intervention arm (adjusted mean: 1665.50 ng / L) than in placebo group (adjusted mean: 1361.90 ng /L) (*P*-value = 0.012). On the other hand, ANCOVA adjusted for baseline levels and the confounding factors showed that supplementation with 2000 IU/d of vitamin D prevented the increment in mean IL-17 serum level in the intervention group compared to the placebo group (adjusted means: 37.84 and 70.09 ng /L, respectively) (*P*-value = 0.039).

**Table 2:** Changes in serum levels of Treg/Th17 related cytokines before and after supplementation with vitamin D or placebo in episodic migraine patients

Variable	Vitamin D Group (***n*** = 38)		Placebo Group (***n*** = 36)		***P***-value
	Baseline	After 12 Weeks	Baseline	After 12 Weeks	
**Serum TGF-β** (ng/L)					
Mean (SD)	1308.75 (382.13)	1623.14 (533.81)	1415.56 (312.24)	1445.86 (424.77)	0.123 ^a^
Adjusted Mean (95%CI) ^b^		1665.50 (1505.95–1825.05)		1361.90 (1191.37–1532.43)	0.012 ^c^
**serum IL-17** (ng/L)					
Mean (SD)	44.21 (67.68)	46.20 (77.32)	66.71 (86.95)	74.99 (85.51)	0.133 ^a^
Adjusted Mean (95%CI) ^b^		37.84 (17.27–58.41)		70.09 (48.08–92.10)	0.039 ^c^

*P* value < 0.05 was considered significant

^a^Independent sample t-test

^b^ After 12 Weeks

^c^ANCOVA test adjusted for baseline levels, age, sex, body mass index (BMI) change, the years having headache, and time from last attack

**Fig. 1:**
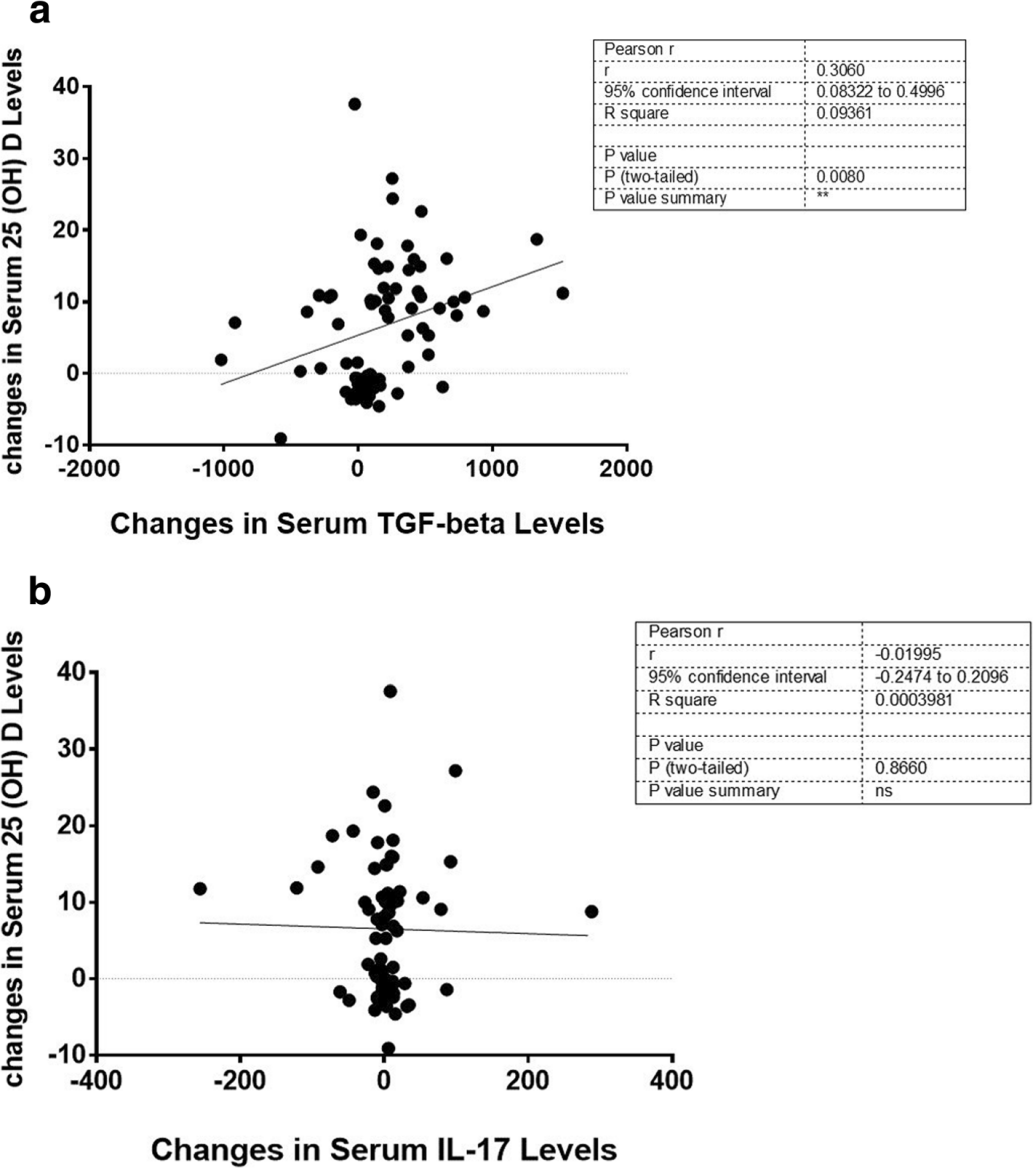
The correlation between changes in serum 25(OH) D and serum levels of Treg/Th17 related cytokines throughout the 12-week trial. **a** The correlation between changes in serum 25(OH) D and serum levels of TGF-β throughout the 12-week trial. **b**. The correlation between changes in serum 25(OH)D and serum levels of IL-17 throughout the 12-week trial

**Fig. 2:**
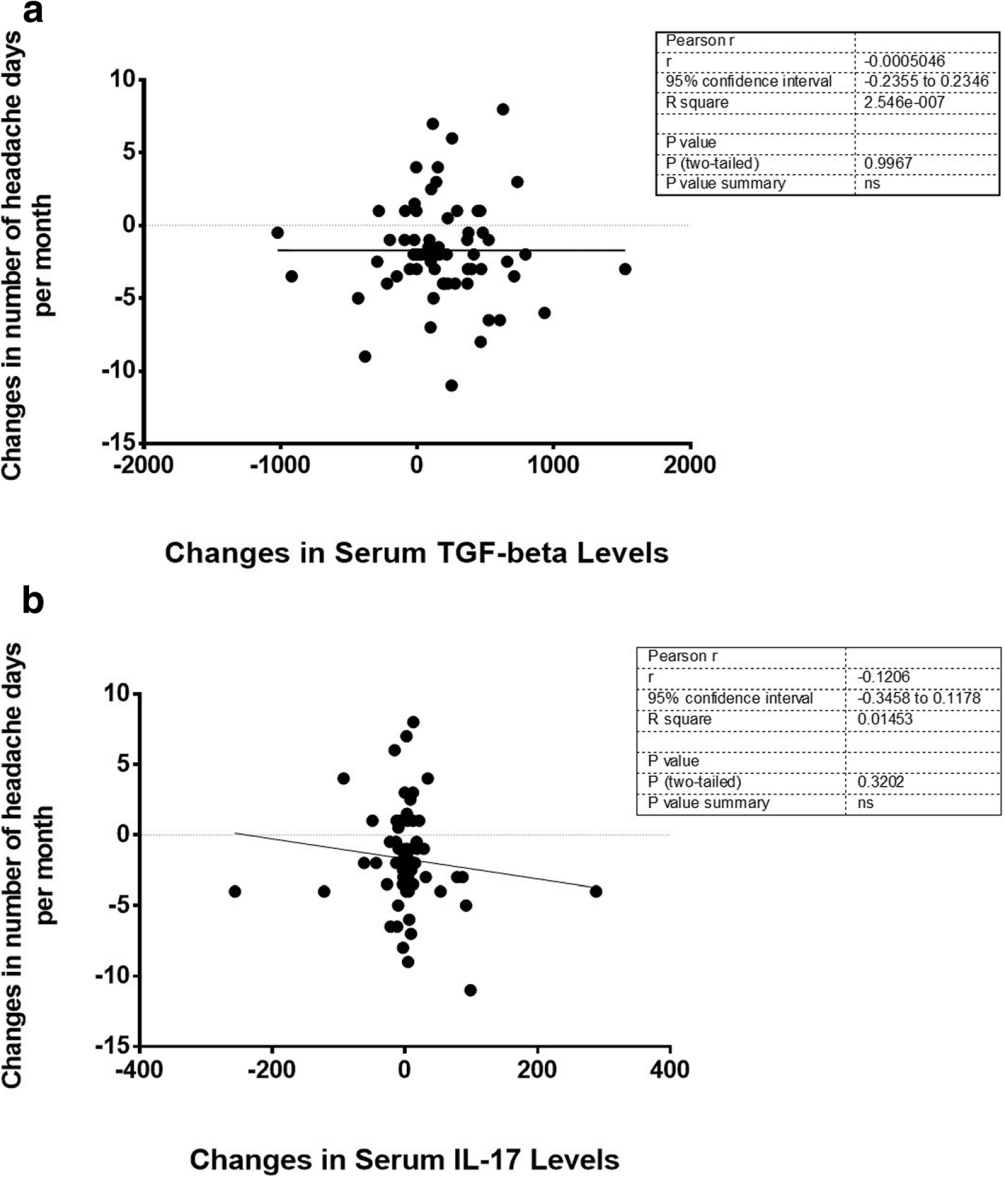
The correlation between changes in the number of headache days per month and serum levels of Treg/Th17 related cytokines throughout the 12-week trial. **a** The correlation between changes in the number of headache days per month and serum levels of TGF-β throughout the 12-week trial. **b**. The correlation between changes in the number of headache days per month and serum levels of IL-17 throughout the 12-week trial

Figure 1(a and b) illustrates the correlation between changes in serum levels of 25(OH) D and changes in serum levels of cytokines secreted by Th17/Treg cells during 3 months of supplementation with vitamin D 2000 IU or placebo. A significant positive correlation between changes in serum level of25 (OH) D and TGF-β was revealed using Pearson correlation analysis (*r* = 0.306, *P*-value = 0.008) (Fig. 1a). However, the negative correlation between serum changes vitamin D biomarker and the pro-inflammatory cytokine secreted by Th17 immune cells, i.e., IL17, was not statistically significant (Fig. 1b). Considering migraine related outcomes, the Pearson correlation analysis failed to show significant results on the correlations between changes in serum IL-17 and TGF-β and changes in number of headache days per month after 12-week of supplementation with 2000 IU/d of vitamin D or placebo (Fig. 2 a and b).

## Discussion

According to current results, it was revealed that in comparison with placebo group, the intervention group who received 2000 IU/day vitamin D3 had higher TGF-β following the 12-week trial. Supplementation with vitamin D cloud also prevent the increment in IL-17 serum level. The positive correlation between changes in serum 25(OH) D levels serum TGF-β concentration reflects that an increase in serum levels of 25(OH) D might be associated with an increment in serum changes of this Treg related cytokine. Thus, our study showed that modulation of Th17/Treg cell balance could be helpful in the improvement of migraine related pathways. It can also be suggested that present findings may provide a theoretical basis for the beneficial effects of vitamin D supplementation in patients with migraine.

Although, to the best of our knowledge, there is not any similar study performed on the effects of vitamin D on Th17/Treg balance in migraine, perhaps the nearest research to that reported in the current paper were the trials in which the effects of this vitamin in MS patients were investigated. Interestingly, it has been shown that supplementation with vitamin D in various dosages (1000 IU/day [38, 39] to 20,000 [40] or 50,000 IU/week [41]) was associated with increased levels of IL-10 [38, 41] and TGF-β [38–40] and on the other hand, reduced concentration of IL-2 [38], IL-17A and IL-6 [41] in MS subjects. Moreover, it has been reported that vitamin D administration could result in increasing TGF-β genes expression [42] and IL-10 levels and declining Th17-associated cytokines (i.e., IL-1β, IL-6, IL-17, IL-22) in EAE [42] and MS patients cell cultures [43]. Although these results seem partially similar to ours; there, however, are still some controversies [44–47].

The immune-protective effects of vitamin D in migraine can be explained after taking into account migraine pathogenesis. Various vasoactive peptides (e.g., CGRP, PACAP, and substance P (SP)) and inflammatory mediators (e.g., nitric oxide (NO), IL-1β, and TNF-α) which are proposed to be involved in migraine pathogenesis, could impose their effects mainly through inducing neuroinflammation induction in the CNS [12, 48–52]. It has been also indicted that imbalance in immune cells including Th1, Th2, Th17 and Treg lymphocytes might play a pathogenic role in inflammatory disorders including migraine, though the involved mechanisms are still not well-understood [11, 17, 18]. Treg and dendritic cells produce IL-10 and TGF-β which, depending on cell type or other conditions, are considered pleiotropic factors but mostly known as suppressive cytokines that incorporate toward the inhibitory actions of regulatory T cells and are involved in preserving immune homeostasis [11, 14]. With respect to this, while the pathogenic potential of TGF-β and IL-17 might mainly depend on the inflammatory context, and specific cells they are produced from, it seems enhanced TGF-β levels and attenuated IL-17 concentration may be of significance in the treatment of migraine [17, 53–55]. Therefore, based on the available shreds of evidence, it can be hypothesized that elevated IL-17 and IL-6 [11] and on the other hand, reduced TGF-β and IL-10 levels might be involved in migraine pain pathophysiology certainly through exacerbating inflammatory responses. In this regard, the possibility of targeting them as a therapeutic strategy against autoimmune and inflammatory disorders progression may still be a contentious concept to many researchers that would benefit from further studies [14].

According to current results and our previous findings [10], it has been shown that supplementation with vitamin D resulted in a significant increase in TGF-β and a slight, non-significant increase in IL-10 levels. Enhanced levels of these cytokines following treatment with vitamin D might lead to attenuating pain initiation in migraine especially through inhibiting immune responses caused by toll-like receptors (TLRs) stimulation [14]. It has been explained that TGF-β and IL-10 in synergy, but not alone, could suppress activation of B cells induced by stimulation of TLRs [14]. Additionally, we demonstrated that the supplementation with vitamin D prevented the increment in the levels of serum IL-17 following the trial compared to placebo group. It has been suggested that IL-17 could stimulate the secretion of different pro-inflammatory factors, particularly NO [53–55]. As the role of NO in migraine attack initiation is well-known [12, 48–50], increased production of this factor might be a linkage between IL-17 and pathological mechanisms involved in this type of headache. Of note, TGF-β seems not to be able to stimulate the differentiation Th17 directly; instead, it should act in synergy with IL-6. Therefore, the role of IL-6 in inducing Th17 cells to generate IL-17 might be more prominent than TGF-β [17, 32]. Since we previously found that within the same trial, with increasing serum 25(OH) D levels, serum concentrations of IL-6 reduced throughout the study [10], the prevention of increase in IL-17 levels in response to vitamin D supplementation might occur following serum IL-6 reduction.

Further, the presence of 1,25(OH)2D in immune cells and VDR in the related genomic regions may additionally confirm the immune-modulatory effects of vitamin D [26, 27]. In addition to the impact of this metabolite on gene expression of inflammatory and immune mediators (i.e., TNF-α in T-cells) and signaling pathways, VDR/RXR interaction with a number of transcription factors, mainly NF-κB, nuclear factor of activated T-cells (NFAT) or glucocorticoid receptor (GCR) have been well-studied before. NF-κB and NFAT activation inhibition by 1,25(OH)_2_D could result in decreased pro-inflammatory mediators’ formation [27, 56–58].

This study had a double-blinded, placebo-controlled design, enrolled definite cases of EM according to ICHDIII criteria [30] and based on headache-specialist neurologists examination, and applied vitamin D 2000 IU which is a safe and available supplement. To reduce the effects of confounders, we did not include the subjects with a history of comorbidities or medications/supplements with a likely effect on vitamin D absorption and functions. We also tried to consider various factors with probable confounding role in the effects of vitamin D using the ANCOVA tests. Further, we asked the patients to not to change their usual medications during the study. However, given that it was not ethical to perform a trial on patients not using any anti-migraine medications, the possible confounding effects of the consumed drugs could not be ruled out. Besides, there might be additional factors that we failed to control for. Thus, it would be most informative if the future studies measure changes in T cell subpopulations in circulation and the gene expression of immune system mediators such as FOXP3, RORγT, IL-1, IL-6, TNF-α, IL-17 and TGF-β following vitamin D supplementation in episodic and chronic migraine patients. Once well-studied and confirmed, the pro-inflammatory/anti-inflammatory cytokines levels ratio can even prove as beneficial serological indicators for monitoring migraine progression, as well as to predict response to the applied treatment. Moreover, due to the lack of data regarding the effects of novel medications on the balance of Th17/Treg, it seems necessary to perform further research on identifying the most effective agents contributing to modulating this balance.

## Conclusion

Based on the results of this study, it was revealed that 12-week vitamin D3 supplementation (2000 IU/day) could enhance the Th17/Treg related cytokines balance in episodic migraine patients. It was also observed that with increasing serum 25(OH) D levels, serum TGF-β concentration tended to increase throughout the trial. These results propose that vitamin D supplementation may help avert the progression of migraine and might be a beneficial complementary approach. Although these findings are promising regarding immune protective effects of vitamin D in migraine, it is needed to be extended and corroborated in further well-designed trials.

## Data Availability

The datasets of the current study are available from the corresponding author on reasonable request.

## Abbreviations

TGF-β: transforming growth factor-beta
IL: interleukin
GBD: global burden of disease
EM: episodic migraine
CM: chronic migraine
CSD: cortical spreading depression
CGRP: calcitonin gene-related peptide
PACAP: pituitary adenylate cyclase-activating peptide
Th: T helper
Treg: regulatory T cells
RORγT: retinoid orphan receptor
Foxp3: forkhead box P3
TNF-α: tumor necrosis factor- α
CRP: C-reactive protein
NF-κB: nuclear factor-κB
EAE: experimental autoimmune encephalomyelitis
MS: multiple sclerosis
iNOS: inducible nitric oxide synthase
25(OH)D: 25-hydroxy-vitamin D
BMI: body mass index
ICHDIII: International Classification of Headache Disorders, 3rd edition criteria
ELIZA: enzyme-linked immunosorbent assay
ANCOVA: analysis of covariance
SD: standard deviation
CI: confidence interval
VDR: vitamin D receptor
NO: nitric oxide
SP: substance P
NOS: NO synthase
TLRs: toll-like receptors
HMGB1: high mobility group box 1
HSP70: heat-shock protein 70
NFAT: nuclear factor of activated T-cells
GCR: glucocorticoid receptor
IFN-γ: interferon-γ
